# A rare case: Spontaneous gastric fistula from a hydatid cyst of the liver

**DOI:** 10.1259/bjrcr.20210087

**Published:** 2022-03-09

**Authors:** Behyamet Onka, Fatima Zohra Benmoula, Wend-Yam Mohamed Traore, Daoud Ali Mohamed, Selma Khouchoua, Ittimade Nassar, Nabil Moatassim Billah

**Affiliations:** 1 Department of Central Radiology, University Hospital Center IBN SINA, Mohamed V University, Faculty of Medicine, Rabat, Morocco; 2 Department of Visceral Surgery: Surgery A, University Hospital Center IBN SINA. Mohamed V University, Faculty ofMedicine, Rabat, Morocco

## Abstract

The hydatid cyst is a worldwide anthropozoonosis, which constitutes a health issue in Northern Africa. It may involve any organ, but it mostly affects the liver. This often asymptomatic disease can lead to multiple complications. Among them, spontaneous fistulization of a hepatic hydatid cyst in the stomach is exceptional even in endemic countries. We report the case of a 38-year-old female with febrile biliary colics due to a hydatid cyst of the liver fistulized in the stomach. The diagnosis was established based upon different clinical, biological and mainly radiological features. She received surgical treatment with satisfactory postoperative outcome.

## Clinical presentation

We report the case of a 38-year-old female with no prior medical history, no comorbidities nor allergies. She presented to the emergency room with a 3-month history of isolated biliary colics. She experienced worsening of the symptoms 1 week prior, with the onset of intermittent fever and vomiting containing membranes. Physical examination found a patient with vital signs within the normal range aside from mild tachycardia, a fever of 38.5°C (Reference value: 36 ° −37°C), no conjunctival pallor, and normally colored urine and stools. Abdominal examination was notable for a distended abdomen and right hypochondrium tenderness. The rest of the physical examination was normal. Laboratory analysis found high levels of C-reactive protein at 240 mg / l (Reference value less than 10 mg / l) and normal white blood cell count at 10,000/mm3 (Reference value: 4000–10000/mm3). Cholestasis assessment was normal: total bilirubin at 4.2 mg / l (Reference value less than 12 mg / l) with a unconjugated fraction at 1.7 mg / l (Reference value less than 2 mg / l), aspartate aminotransferase at 37 IU / l (Reference value: 8–30 IU / l), alanine aminotransferase at 30 IU / l (Reference value: 6–25 IU / l).

## Differential diagnosis

All the causes of gallstones complications (acute cholecystitis and cholangitis), liver abscess, pancreatitis but also causes of febrile right upper quadrant abdominal pain : pyelonephritis, subhepatic appendicitis, or even right basal pneumonia.

## Imaging findings

**Figure 1.**
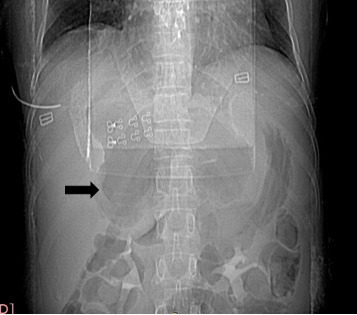
X-ray of the abdomen without preparation: Large extra luminal gas collection (black arrow).

**Figure 2.**
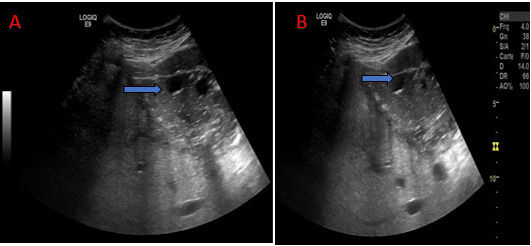
Abdominal ultrasound: Multi loculated cystic mass of the left lobe, stage III of Gharbi (blue arrow).

On the erect plain abdominal film, there was an abnormal extra luminal gas collection in the epigastric region (Figure 1). An abdominal ultrasound showed a multi loculated cystic mass adjacent to the left liver lobe, and the stomach; consistent with a type III of Gharbi hydatid cyst (Figure 2A and B).

A computed tomography scan of the chest and abdomen was performed with intravenous contrast demonstrating a cystic mass within the left liver lobe parenchyma, with an air fluid level, well-defined and thick capsule, compressing the stomach. Further assessment revealed a close contact with the gastric wall and an air filled communication track with the cyst.

**Figure 3.**
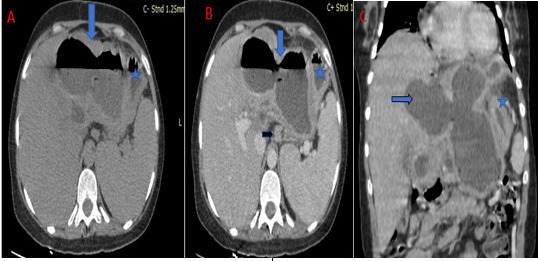
Abdominal CT scan in axial (**A, B**) and coronal (**C**) sections without intravenous contrast media: Large cystic mass of the left liver with thickened wall, and air fluid level (blue arrow) next to the stomach (blue star) with a lymphadenopathy (black arrow).

**Figure 4.**
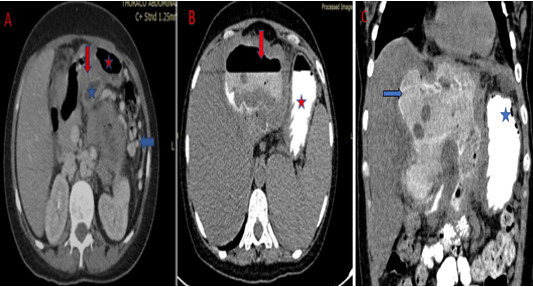
Abdominal CT scan in axial (**A**) sections with intravenous contrast media: Visualization of a fistula (red arrow) between the cyst (blue star) of the left liver and the stomach (red star). It is associated with compression of the splenic vein and collateral venous circulation (blue arrow). After oral contrast administration (abdominal CT in axial (**B**) and coronal (**C**) section): Visualization of contrast product in the cyst.

In addition we observed mass effect on the splenic vein resulting in many collateral vessels and a markedly dilated coronary vein. The CT also revealed coelio mesenteric lymphadenopathies (Figures 3 and 4A).

Finally, there was no pulmonary or mediastinal abnormalities on the chest CT examination.

**Figure 5.**
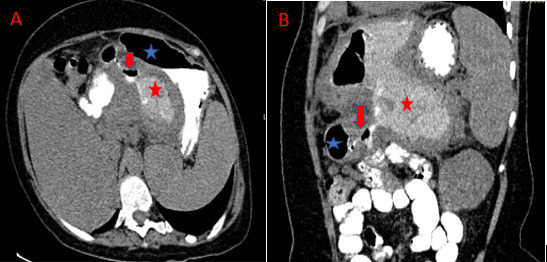
Abdominal CT after axial (**A**) and coronal (**B**) oblique reconstructions and oral contrast administration: Visualization of the fistula track (red arrow) between the stomach (blue star) and the cyst with passage of the contrast agent into the cyst (red star).

We then chose complete the CT examination with oral contrast to confirm the presence of an abnormal communication between the liver mass and gastric lumen. It showed the contrast delineating a fistula track between the stomach through the lesser curvature wall and the cyst (Figures 4B, C, and 5).

## Treatment, outcome and follow-up

**Figure 6.**
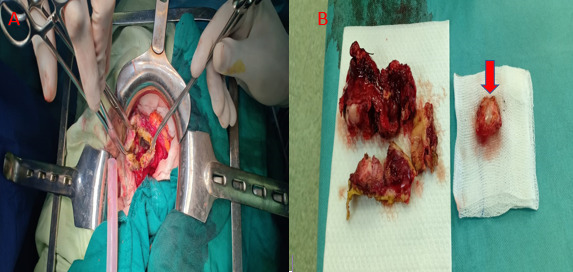
Intraoperative exploration of the cyst and resection of the protruding dome +wedge gastric resection. Image of girl gallbladder (red Arrow).

The patient underwent a laparotomy, finding an enormous multilocular hepatic hydatid cyst adherent to the gastric wall. Cystic exploration found of a fistula between the cyst and the stomach (Figure 6).

A nasogastric tube was placed, and aspirated purulent fluid containing membranes, suggesting the presence of a fistula. A resection of the protruding dome was performed with a gastric wedge resection and the stomach defect was sutured. Finally a surgical drain was placed in the residual cavity.

Post-operative outcome was satisfactory and the patient was discharged after 3 weeks.

## Discussion

Fistulization of the hydatid cyst in the gastro intestinal (GI) tract is extremely rare even in highly endemic countries.,^1,2^ the most common site of perforation being the stomach. To the best knowledge of the other no case report of this nature has been reported.

According to surgical records of hepatic hydatid cysts: 0.29% were ruptured into the gastrointestinal tract and 0.15% into the duodenum. In a multicentric study conducted by Zaouche on hydatid cyst complications in 1994, only 3 cases were ruptured into the hollow viscus.^3–5^

It is believed that hydatid cyst communication with the bowel lumen depends on : cyst location, infection and close contact.

Communication between the hydatid cyst and any part of GI tract (the stomach, duodenum and transverse colon) is more likely to occur when the cyst is located on the inferior surface of the liver.^3,4^

Also, infection of the cyst with the thickening of its walls may create adhesions between the cyst and the surrounding organs.

Moreover, continuous friction by the thickened or calcified cyst results in bowel wall erosion.

These last two conditions were met in our patient (Figures 3 and 4).

On the other hand, this complication is known for its clinical polymorphism ranging from abdominal discomfort to pain, or fever.

Typically, the presence of membranes in the stools “hydatidorrhea” or in the vomit “hydatidemesis” are highly suggestive.^6^

In our case, the patient presented with biliary colics, fever and hydatid vomiting.

Although this complication is hardly ever diagnosed pre operatively, imaging appears to have a key role in making the diagnosis.

Because of its availability and simplicity, ultrasound is the first imaging technique used in an emergency setting.

It helps characterize the cystic nature of the lesion and the Gharbi classification which remains the first and most widely used classification, particularly for ultrasound.^7^

The imaging findings of hydatid disease vary according to its stage of development, ranging from cystic lesions to a completely solid mass. The classification of Gharbi is currently replaced by the World Health Organization (WHO) classification, useful in deciding the appropriate management depending on the stage of the cyst on ultrasound.^8^

There is a correspondence between the two classifications. The cystic echinococcus (CE) one cyst refers to a simple cyst (Gharbi Type I). The CE2 stage consists of a multi vesicular cyst, containing septations, described as a “rosette” or a “honeycomb” sign (Gharbi Type III). This appearance is pathognomonic.

The CE3 cyst contains a daughter cyst within the encompassing parent cyst. The daughter cyst can have detached laminated membranes, giving a “water lily” sign (Gharbi Type II). The CE4 stage has a mixed hypoechoic and/or hyperechoic matrix, with no daughter cysts (Gharbi Type IV). The CE5 cyst is characterized by a thick partially or completely calcified wall (Gharbi Type V). Ultrasound classifications can be extrapolated to computed tomography and magnetic resonance imaging.^9–11^

Cross-sectional imaging appears to be essential in the event of complications particularly for the preoperative assessment.

CT scan plays a major role, showing a cystic lesion of the liver commonly on the inferior surface, with signs of infection like thickening and enhancing wall, containing an air fluid level, and responsible for a mass effect and very intimate contact with the stomach.

Oral contrast administration makes it easier to visualize the fistula between the cyst and the stomach, demonstrated by the passage of the iodine from the stomach to the cyst.^6,12^

This examination protocol was the key to make the diagnosis in our case.

However, the fistula is not always visualized, often related to hydatid membranes obstructing the communication, in which case the diagnosis is only made in the operating room.^13^

Currently, surgery is the standard treatment for complicated cysts as well as extensive, large and symptomatic uncomplicated cysts.

Preoperative medical treatment with albendazole is recommended because it decreases the viability of the cysts. There is little specific data on the benefits of postoperative or perioperative use of medical treatment. But most authors agree on the effectiveness of postoperative medical treatment in reducing the risk of recurrence.^10^

Being an emergency surgery in our case, the patient did not receive premedication.

## Learning points

- Hydatid cyst is a worldwide anthropozoonosis. It mainly affects the liver and presents several complications dominated by fistulas and infection. Fistulization in the bowel lumen remains exceptional and is dominated by fistulization in the stomach.
- Imaging is essential for correct and appropriate diagnosis of gastric cyst fistulization and to avoid treatment delays. The key examination is computed tomography with oral contrast, making it easy to visualize the gastric fistula. Treatment is mainly surgical.
- Early diagnosis and surgical treatment of complicated and symptomatic intra abdominal hydatid cysts reduces morbidity and mortality.
